# Welcome to the next generation of Malaria Rapid Diagnostic Tests: Comparative Analysis of NxTek Eliminate Malaria P.f, Biocredit Malaria Ag Pf, and SD Bioline Malaria Ag Pf for Plasmodium falciparum Diagnosis in Ghana

**DOI:** 10.21203/rs.3.rs-3459263/v1

**Published:** 2023-10-19

**Authors:** Tolulope A Kayode, Agyapong Kofi Addo Addo, Thomas Kwame Addison, Austine Tweneboah, Stephen Opoku Afriyie, Dawood Ackom Abass, Ayesha Seth, Abraham K. Badu-Tawiah, Kingsley Badu, Cristian Koepfli

**Affiliations:** University of Notre Dame; Kwame Nkrumah University of Science and Technology; Kwame Nkrumah University of Science and Technology; Kwame Nkrumah University of Science and Technology; Kwame Nkrumah University of Science and Technology; Kwame Nkrumah University of Science and Technology; The Ohio State University; The Ohio State University; Kwame Nkrumah University of Science and Technology; University of Notre Dame

**Keywords:** Malaria diagnosis, rapid diagnostic test, ultrasensitive RDT, HRP2, LDH

## Abstract

**Background:**

Accurate diagnosis and timely treatment are crucial in combating malaria.

**Methods:**

We evaluated the diagnostic performance of three Rapid Diagnostic Tests (RDTs) in diagnosing febrile patients, namely: Abbott NxTek Eliminate Malaria Ag Pf (detecting HRP2), Rapigen Biocredit Malaria Ag Pf (detecting HRP2 and LDH on separate bands), and SD Bioline Malaria Ag Pf (detecting HRP2). Results were compared to qPCR.

**Results:**

Among 449 clinical patients, 45.7% (205/449) tested positive by qPCR for *P. falciparum* with a mean parasite density of 12.5parasites/μL. The sensitivity of the Biocredit RDT was 52.2% (107/205), NxTek RDT was 49.3% (101/205), and Bioline RDT was 40.5% (83/205). When samples with parasite densities lower than 20 parasites/uL were excluded (n=116), a sensitivity of 88.8% (79/89, NxTek), 89.9% (80/89, Biocredit), and 78.7% (70/89, Bioline) was obtained. All three RDTs demonstrated specificity above 95%. The limits of detection was 84 parasites/μL (NxTek), 56 parasites/μL (Biocredit, considering either HRP2 or LDH), and 331 parasites/μL (Bioline). None of the three qPCR-confirmed *P. falciparum* positive samples, identified solely through the LDH target, carried *hrp2/3* deletions.

**Conclusion:**

The Biocredit and NxTek RDTs demonstrated comparable diagnostic efficacies and both RDTs performed better than Bioline RDT.

## Background

Malaria remains a significant public health concern in sub-Saharan Africa, including Ghana, where an estimated 5.3 million cases and 12,500 deaths were reported in 2021 [1]. Rapid and accurate diagnosis of malaria is crucial for effective treatment and control of the spread of the disease [2]. Rapid diagnostic tests (RDTs) are immunochromatographic assays widely used for malaria diagnosis, particularly in resource-limited settings. RDTs are easy to use, require minimal training, and provide rapid results [3]. High sensitivity of RDTs is crucial to detect low-density infections.

RDTs for *Plasmodium falciparum* rely on detecting specific proteins such as histidine-rich proteins 2 and 3 (HRP2 and HRP3), parasite lactate dehydrogenase (LDH), or aldolase [4]. HRP2-based RDTs are considered the most sensitive [5–8]. However, deletions in the *hrp2* and *hrp3* genes will lead to false-negative RDT results, even in patients with high parasite density infections [4, 9]. These deletions have been observed in various countries, in particular in east Aftica [10–12], but also at low frequencies in Ghana [13].

Numerous studies have investigated the diagnostic performance of RDTs and found varying sensitivities [5–9, 14–18]. Variation in sensitivity can be as a result of differences in RDT design, characteristics of the study population (e.g. clinical vs. subclinical infections, or differences in age groups reflecting different levels of acquired immunity and thus different parasite densities), choice of the gold standard (e.g., microscopy or PCR), and differences among sample processing and PCR assays resulting in variation of the limit of detection and parasite quantification by qPCR [3, 17, 19, 20, 21]. As a result, data on sensitivity and LOD of RDTs tested using different protocols are difficult to compare.

Here, we assess the performance of the NxTek Eliminate Malaria Ag Pf, SD Bioline Malaria Ag Pf, and Biocredit Malaria Ag Pf RDTs in diagnosing clinical patients in Ghana. The NxTek and Biocredit test are considered next-generation ultra-sensitive tests. The NxTek and Bioline have one test band for HRP2. The Biocredit Malaria Ag Pf. (LDH/HRP2) has two separate test bands for HRP2 and LDH. Having both targets as separate bands allow diagnosis in the case of *hrp2/3* deletion and enables surveillance of deletion status, as samples positive for LDH but negative for HRP2/3 can be selected for molecular confirmation of deletion status.

## Methods

### Ethical approval

Prior to sample collection, informed written consent was obtained from each individual. For minors, assent was obtained in addition to consent obtained from legal guardians. This study was approved by the Committee on Human Research, Publications, and Ethics of the School of Medical Sciences, KNUST (CHRPE/AP/030/20), the University of Notre Dame Institutional Review Board (19–04-5321), and The Ohio State University Institutional Review Board (2020H0539).

### Study site and sample collection

Samples were collected from health centers in Mankranso (6.8181° N, 1.8635° W) and Agona (6.9347° N, 1.4870° W) in the Ashanti region of Ghana. The Ashanti region has a reported malaria prevalence of 22% by microscopy [22]. The samples were obtained during the rainy seasons, between August and September 2022, known to be periods of high malaria transmission [23]. Blood samples (approximately 2 mL) from participants were collected in EDTA tubes, and malaria screening with the three RDTs was performed on-site. Study participants were treated as per the national guidelines by healthcare providers at the hospital.

### Rapid diagnostic tests kits and testing

Three different RDT kits were compared, the RDT, NxTek Eliminate Malaria Ag Pf. ((lot no. 05LDG008B), manufactured by Abbott, the Bioline Malaria Ag Pf. (lot no. 05CDH037C), also manufactured by Abbott, and the Biocredit Malaria Ag Pf. (LDH/HRP2) (lot no. H052BSA002), manufactured by Rapigen. Test were conducted according to manufaturer’s instructions. Tests were considered invalid and repeated if the control band was not positive.

### DNA extraction, *var*ATS qPCR, and *hrp2/3* deletion typing

DNA was extracted from 100 μL blood and eluted in 100 μL elution buffer using the Macherey-Nagel NucleoMag extraction kit. qPCR of the *P. falciparum var*ATS multi-copy gene was carried out using a previously described protocol with 4 μL DNA as target resulting in a 95% limit of detection of 0.3 parasties/μL blood [24]. *hrp2/3* deletion typing for samples that were negative for HRP2 on RDTs but positive for LDH and that were confirmed positive by qPCR was done by multiplexed digital PCR as previously described [25].

### Data analysis

We assumed a test positivity of 50% and obtained a minimum sample size of 384 required to detect a 5% difference in sensitivity with a 95% confidence level (α = 0.05, power 1 – β = 0.20) using a previously described method [26]. A total of 449 clinical samples were obtained, exceeding the minimum requirement. Performance characteristics were determined for all three RDTs, and for the Biocredit RDT, the HRP2 and LDH targets were considered separately and in combination [3].

Sensitivity was calculated as the number of infections detected by an RDT divided by the number of infections detected by qPCR, and against thresholds of 2000, 200, and 20 parasites/μL (by qPCR). This was done to comparability with other studies, as different methods for sample collection, DNA extraction, and qPCR result in different sensitivities and, thus, different numbers of positive samples [21]. Specificity was calculated as the proportion of negative RDTs among individuals that tested negative by qPCR. The positive predictive value (PPV) was calculated as the probability that the infection is present when the RDT is positive and parasite density is >20 parasites/μL [27]. Samples with densities of >0 to 20 parasites/μL were exluded from the calculation of NPV and PPV. This threshold was set as low-density infections might be incidental, i.e. likely are not the cause of febrile illness. The negative predictive value (NPV) was calculated as the probability that qPCR is negative when the RDT is negative [27]. The limit of detection (LoD) was defined as the lowest parasite density where a qPCR-positive infection would be detected with 95% probability and logistic regression analysis was conducted to determine the LoD of each RDT target.

The area under the receiver operating characteristic curve (AUC) was calculated with a nonparametric analysis using 1000 bootstrap replications. As for PPV, a threshold of 20 parasites/μL by qPCR was set to count a sample as true positive, and samples with >0 to 20 parasites/μL were exluded from the calculation. As parasite density distributions were skewed, geometric mean densities are given whenever densities are reported. CI95 stands for the 95% confidence interval. The *p* values to compare groups for qPCR test positivity and RDT sensitivity were calculated by Chi-square test, while Welch’s ANOVA was used for parasite density.

## Results

### Study population demographics

A total of 449 clinical samples were collected and analyzed. Table 1 provides the demographic information of the study participants. Among the participants, only 7.8% were below 5 years of age, while the majority (67.5%) were above 15 years of age. The majority of participants were female (71.7%).

### RDT diagnostic performance

205/449 (45.7%) clinical samples tested positive for *P. falciparum* by varATS qPCR, with a mean parasite density of 12.5 parasites/μL. There were no statistically significant differences in positivity by qPCR based on participant’s age or gender (Table 1). Of the 205 qPCR-positive samples, 142 (69.3%) had densities <200 parasites/μL blood. There was no significant difference in densities between male and female participants (p=0.44, Table 1). Parasite density was highest in participants aged 0 to 5 (Table 1).

Table 2 shows the sensitivity and specificity of the evaluated RDTs. The NxTek and Biocredit RDTs (considering either the HRP2 or LDH band) showed similar sensitivity, detecting 49.3% (101/205) and 52.7% (108/205) of qPCR-confirmed infections. The Bioline RDT had lower sensitivity, detecting 39.5% (81/205) of qPCR-positive infections. As the threshold for parasite density decreased (from >2000 parasites/μL to >200 parasites/μL to >20 parasites/μL), the sensitivity of the RDTs also decreased (Table 3). All RDTs demonstrated specificity levels above 95% (Table 2). The limit of detection (LoD) was determined as 84 parasites/μL (CI95: 40, 177) for the NxTek RDT, 56 parasites/μL (CI95: 28, 118) for the Biocredit RDT (considering either the HRP2 or LDH target), and 331 parasites/μL (CI95: 148, 739) for the Bioline RDT (Table 2). The Negative Predictive Value (NPV) was 95.9% (CI95: 92.9, 97.7) for the NxTek RDT, 96.3% (CI95: 93.4, 97.9) for the Biocredit RDT, and 92.7% (CI95: 89.4, 94.9) for the Bioline RDT. All RDTs achieved a test accuracy (area under the curve (AUC)) of >0.85 (Table 2).

### Comparison of HRP2 vs. LDH, and *hrp2/3* Deletion Typing

**Figure 1:**
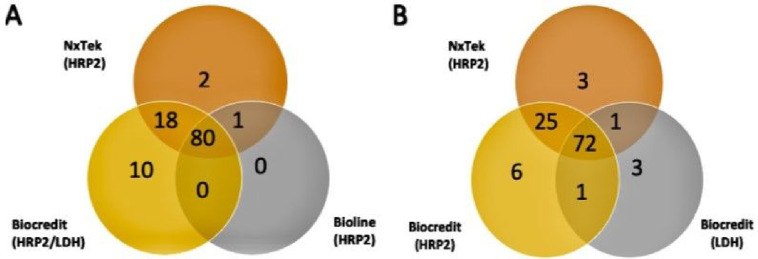
(A) The detection of qPCR-confirmed *P. falciparum* infections using three rapid diagnostic tests (RDTs): Nxtek (HRP2), Biocredit (HRP2/LDH), and Bioline (HRP2). (B) Comparison between the Nxtek and Biocredit RDT kits to accurately detect true positive *P. falciparum* infections with HRP2 and LDH targets. A total of 111 out of 205 qPCR-confirmed infections were detected by these RDTs as true positive tests.

The Biocredit RDT demonstrated higher sensitivity for the HRP2 target (88.8% at densities >20 parasites/μL), compared to the LDH target (75.3%). Three qPCR-confirmed infections were detected by LDH only (Figure 1) thus, sensitivity for HRP2 only was minimally lower compared to when both HRP2 and LDH targets were considered (Table 2). The NPV for the HRP2 target was slightly higher (95.9%, CI: 92.9, 97.7) than the LDH target (91.6%, CI: 88.4, 94.0). None of the three samples positive by LDH but negative by HRP2 carried *hrp2* or *hrp3* deletions. The parasite densities of these samples ranged from 2–5 parasites/μL.

## Discussion

This study showed similarly high sensitivities of the NxTek and the Biocredit RDT. These tests are considered ultra-sensitive, i.e. substantially more sensitive than previous tests such as the SB Bioline. They detected around 50% of all qPCR-positive infections, compared to around 40% by the SD Bioline. At densities >200 parasites/μL, the NxTek and Biocredit sensitivity was >93%, and 89% for the SD Bioline. The LOD of the NxTek and Biocredit is approximately four-fold lower than the LOD for the SD Bioline, and the LOD of the Biocredit confirmed values obtained from a previous study in Burundi applying the same methodology [3]. RDT sensitivity reached 73% in children aged 6–15 years. Older participants (> 15 years) had lower parasite densities, resulting in lower RDT sensitivity.

None of the three *P. falciparum* malaria-positive samples detected via the LDH target alone carried *hrp2/3* deletions. The current data thus corroborated recent findings of very low frequency of *hrp2/3* deletions in Ghana [28, 29]. The diagnostic performance of the LDH target in the Biocredit RDT was found to be comparable to that of the conventional Bioline HRP2 RDT. This suggests that the Biocredit RDT, with its LDH target, can be a suitable alternative to the Bioline RDT in regions where *hrp2* deletion is prevalent. While to-date in Ghana HRP2-based RDTs remain effective, the Biocredit RDT will be a reliable option for malaria diagnosis shall deletions ever spread in the country.

In conclusion, the NxTek and Biocredit RDTs showed higher sensitivity and a lower LOD compared to the SD Bioline RDT, which has been in use for clinical diagnosis by the Ghana National Malaria Control Programme [30]. Such more sensitive RDTs are crucial to administer proper treatment and to accelerate malaria elimination efforts.

## Figures and Tables

**Table 1.** Demographics of the study population, parasite density, test positivity (by qPCR) and RDT sensitivity by age group and gender.

Parameter	Category	N	qPCR Test positivity	p	Parasite density [95CI]	p	RDT sensitivity^1^	p
**Age (years)**	0 to 5	35 (7.8%)	48.60% (17/35)		78.85 [75.91, 81.87]		70.1% (12/17)	
	6 to 15	111 (24.7%)	48.80% (52/111)	0.87	41.29 [40.05, 42.53]	0.14	73.08% (38/52)	<0.01
	> 15	303 (67.5%)	44.90% (136/303)		6.28 [5.90, 6.67]		44.85% (61/136)	
**Gender**	Male	127 (28.3%)	49.60% (63/127)		12.38 [11.21, 13.56]		47.62% (30/63)	
	Female	322 (71.7%)	44.01% (142/322)	0.58	12.55 [11.48, 13.62]	0.44	57.04% (81/142)	0.49
	Total	449 (100%)	45.66% (205/449)		12.5 [11.21, 13.62]		54.15% (111/205)	

1 For the RDT data, the results from all three RDTs were combined, with either RDT and either target (HRP2 or LDH) positive counting as a positive test.

**Table 2.** Measure of diagnostic performance of Bioline, NxTek and Biocredit RDTs

Diagnostic Measure	Bioline HRP2 [95CI]	NXTek HRP2 [95CI]	Biocredit HRP2 [95CI]	Biocredit LDH [95CI]	Biocredit HRP2/LDH [95CI]
Sensitivity (all densities)	39.5% [32.8,46.6]	49.3% [42.2,56.3]	50.7% [43.7, 57.8]	37.6% [30.9, 44.6]	52.7% [45.6, 59.7]
Sensitivity (>2000 parasites/μL)	94.6 [81.8, 99.3]	100 [94.3, 100]	94.6 [81.8, 99.3]	97.3 [85.8, 99.9]	97.3 [85.8, 99.9]
Sensitivity (>200 parasites/μL)	88.9 [78.4, 95.4]	95.2 [86.7, 99.0]	92.1 [82.4, 97.4]	92.1 [82.4, 97.4]	93.7 [84.5, 98.2]
Sensitivity (>20 parasites/μL)	78.7 [68.7, 86.6]	88.8 [80.3, 94.5]	88.8 [80.3, 94.5]	75.3 [65.0, 83.8]	89.9 [81.7, 95.3]
Specificity	98.8% [96.5, 99.8]	97.1% [94.2, 98.8]	97.1% [94.2, 98.8]	98.8% [96.5, 99.8]	96.3% [93.1, 98.3]
Positive Predictive Value	95.9% [88.3, 98.6]	91.9% [84.4, 95.9]	91.9% [84.4, 95.9]	95.7% [87.8, 98.6]	89.9% [82.3, 94.4]
Negative Predictive Value	92.7% [89.4, 94.9]	95.9% [92.9, 97.7]	95.9% [92.9, 97.7]	91.6% [88.4, 94.0]	96.3% [93.4, 97.9]
Accuracy (AUC)	0.887 [0.84, 0.93]	0.929 [0.89, 0.96]	0.929 [0.89, 0.96]	0.870 [0.82, 0.92]	0.931 [0.90, 0.96]
95% LOD (parasites/μL)	331 [148, 739]	84 [40, 177]	85 [43, 171]	349 [142, 858]	56 [28, 118]

## Data Availability

All data is provided in supplementary File S1.
